# Feasibility and outcomes of robotic colorectal cancer surgery in patients with high body mass index

**DOI:** 10.1007/s10151-025-03278-1

**Published:** 2026-03-07

**Authors:** C. Chew, A. Panesa, M. U. Haq, E. Gilbert-Kawai, S. Ahmed

**Affiliations:** 1 Department of Colorectal Surgery, Liverpool University Hospital NHS Foundation Trust, University of Liverpool, Prescot Street, Liverpool, L7 8XP UK; 2 Department of Molecular and Clinical Cancer Medicine, University of Liverpool, Liverpool, L69 7BE UK; 3 Department of Anaesthesia and Critical Care, Liverpool University Hospital NHS Foundation Trust, Prescot Street, Liverpool, L7 8XP UK

**Keywords:** Body mass index, Robotic surgical procedures, Colorectal neoplasms, Minimally invasive surgery, Perioperative care

## Abstract

**Background:**

Minimally invasive techniques are the standard of care in colorectal surgery. However, high body mass index (BMI) presents technical and anaesthetic challenges. Robotic-assisted surgery offers potential advantages in this population; however, given its relatively recent adoption, outcome data remain limited. This article presents a single-centre case series evaluating the short-term surgical and anaesthetic outcomes of obese patients undergoing robotic colorectal resections.

**Methods:**

A retrospective review was performed of patients with BMI > 30 kg/m^2^ who underwent robotic colorectal cancer resection at Liverpool University Hospital NHS Trust between July 2019 and April 2024. Demographic, surgical, anaesthetic, and clinical outcome data were collected from a prospectively maintained database. Key measures included operative time, Trendelenburg positioning, critical care admissions, complications, and mortality.

**Results:**

Seventy-five patients [mean BMI 35.8 (range 30.0–66.1) kg/m^2^] underwent robotic resection. Conversion to open surgery occurred in one case (1.3%). Mean operative time was 380 min for rectal and 289 min for colonic resections. The average Trendelenburg tilt was 20^o^ (range 15–22), with an average lateral tilt of 15^o^ (range 10–20), and mean head down duration was 225 (range 120–320) min. Fifty-six (74.7%) patients had primary anastomosis; the anastomotic leak rate was 9.3%. Thirty-two per cent of patients were electively admitted to the critical care as part of our institutional pathway for high BMI-cases. Of these, three patients (4.0%) required overnight mechanical ventilation and were both extubated the following morning without further organ support. One case of postoperative visual disturbance, attributed to positioning, resolved fully. The 30- and 90-day mortality rate was 1.3%.

**Conclusion:**

Robotic colorectal surgery is a feasible and safe option in elevated BMI patients, with low conversion rates and acceptable morbidity. Importantly, prolonged Trendelenburg position did not appear to increase anaesthetic risk, with only one reversible positioning-related complication observed.

## Introduction

Minimally invasive techniques have become the standard of care in colorectal surgery due to their benefits, including reduced postoperative pain, faster recovery, and shorter hospital stays [1–7]. However, rising global obesity rates have introduced new challenges, particularly in the surgical and anaesthetic management of patients undergoing colorectal resection [8]. Obesity is defined by the World Health Organization (WHO) as a body mass index (BMI) of 30 kg/m^2^ or higher, with further subdivisions into Class I (30.0–34.9 kg/m^2^), Class II (35.0–39.9 kg/m^2^), and Class III (≥ 40.0 kg/m^2^) obesity [9]. Obesity is associated with an increased risk of colorectal cancer and poorer treatment outcomes, and performing minimally invasive surgery in this population becomes more complex [10, 11].

Laparoscopic surgery, whilst effective, can be technically demanding in obese patients—especially during pelvic procedures—due to limited space and reduced instrument manoeuvrability [12, 13]. Robotic-assisted surgery offers potential solutions to these limitations, providing enhanced 3D visualisation, improved dexterity through articulating instruments, and a stable platform that facilitates precise dissection in confined spaces. These features may be particularly advantageous in obese patients, in whom pelvic access and visualisation are frequently compromised [14, 15].

Despite these benefits, the adoption of robotic techniques for colorectal surgery has been cautious. Concerns remain regarding longer operative times and the potential anaesthetic complications associated with pneumoperitoneum and the necessity for prolonged Trendelenburg positioning. Steep Trendelenburg positioning can lead to significant physiological changes, including reduced pulmonary compliance, impaired ventilation, increased intracranial and intraocular pressures, airway oedema, and haemodynamic instability [16]. These risks may be amplified in obese patients, and to date there is limited literature guiding safe anaesthetic practices in this setting, particularly regarding the safe duration of steep Trendelenburg positioning.

This case series evaluated the short-term surgical and anaesthetic outcomes of obese patients undergoing robotic colorectal resections. Our aim was to describe the feasibility of this approach in a high BMI population, with particular attention to perioperative morbidity. By reporting both surgical and anaesthetic parameters, we hope to contribute to the growing body of evidence supporting the feasibility and safety of robotic colorectal surgery in this high-risk population.

## Methods

### Study design

The Royal Liverpool Hospital robotic colorectal program was established in April 2012. All patients registered in our prospectively collected Somerset Cancer registry who underwent a colorectal resection between July 2019 and April 2024 were reviewed. Patients were excluded if they underwent surgery for benign conditions, anal cancer, neuroendocrine tumours, primary gynaecological or urological malignancies, or small bowel and appendicular tumours.

Surgical approach was selected based on multidisciplinary assessment of individual patient factors. Open surgery was reserved for cases in which minimally invasive surgery was not feasible, such as those with prior laparotomy, extensive adhesions, or contraindications to pneumoperitoneum or Trendelenburg positioning due to severe cardiopulmonary comorbidities. Allocation was therefore based primarily on physiological suitability rather than tumour complexity.

Patients were allocated to surgeons on a rotational basis according to the institutional referral pathway. There was no preferential allocation of more complex cases to any particular technique. Only one patient was considered unfit for minimally invasive surgery (severe cardiopulmonary limitation) and was therefore assigned to the open cohort. Robotic procedures were performed exclusively by accredited robotic consultants, whereas laparoscopic and open cases were performed by consultants or senior trainees under direct consultant supervision. No robotic cases in this series were performed by trainees.

All open and laparoscopic procedures were performed by consultant colorectal surgeons with at least 5 years’ independent experience in minimally invasive and open techniques. Robotic procedures were performed exclusively by accredited Da Vinci colorectal surgeons, each with > 100 robotic colorectal resections prior to the study period, ensuring consultant-level expertise across all modalities.

Patient demographic data included age, gender, BMI, American Society of Anaesthesiologists (ASA) classification, and comorbidities such as smoking, diabetes, and hypertension. Robotic surgical parameters recorded included robot docking time, the degree of both head-down and lateral table tilt, total operative time, and total head-down time. Anaesthetic data collected comprised critical care admission, overnight intubation, the use of intra- and postoperative vasopressors and/or inotropes, and the type of analgesia administered alongside general anaesthesia (neuraxial, local anaesthetic wound catheters, or local anaesthetic infiltration).

All critical care admissions in this cohort were planned and elective, as per our institutional pathway for high-BMI patients. Clinical outcome data included the length of inpatient stay, complications (such as return to theatre, conversion to open surgery, readmission, and anastomotic leak), and 30- and 90-day mortality rates. Baseline characteristics, including comorbidity profile, ASA class, and clinical staging, were assessed to ensure comparability among robotic, laparoscopic, and open cohorts (Table 1).

**Table 1:** Baseline patient characteristics by surgical technique

Surgical technique	Robotic [*n* (%)]	Laparoscopic [*n* (%)]	Open [*n* (%)]	Level of significance (*p* value)
Sample size	75 (44.1%)	60 (35.3%)	35 (20.6%)	
Gender				
Male	41 (54.7%)	29 (48.3%)	21 (60%)	0.620
Female	34 (45.3%)	31 (51.7%)	14 (40%)	
Age (range)	65 (36–80)	67 (42–81)	69 (36–78)	0.020
American Society of Anaesthesiologists Classification				
I	0 (0.0%)	1 (1.7%)	0 (0.0%)	0.210
II	33 (44.0%)	31 (51.7%)	18 (51.4%)	
III	41 (54.7%)	26 (43.3%)	17 (48.6%)	
IV	1 (1.3%)	2 (3.3%)	0 (0.0%)	
Average BMI (kg/m^2^, range)	35.8 (30.0–66.1)	32.5 (31.1–41.6)	34.5 (30.5–40.8)	0.030
Preoperative T stage				
T1	14 (18.6%)	4 (6.7%)	4 (11.4%)	0.048
T2	19 (25.3%)	18 (30.0%)	6 (17.1%)	
T3	38 (50.7%)	33 (55.0%)	19 (54.3%)	
T4	4 (5.3%)	5 (8.3%)	6 (17.1%)	

*BMI* body mass index, *ASA* American Society of Anaesthesiologists

### Anaesthetic considerations and postoperative complications

At our institution, all patients undergo routine preoperative assessment in a nurse-led clinic, with high-risk cases additionally reviewed by a consultant anaesthetist for optimisation and consideration of elective postoperative critical care admission, which is standard practice at our centre. Intra- and postoperative analgesic techniques, anaesthetic maintenance (volatile or total intravenous), and decisions regarding invasive monitoring were made at the discretion of the individual anaesthetic consultant. When regional analgesia was employed, the transversus abdominis plane (TAP) block was used as standard.

Patients were positioned in line with institutional practice, catheterised, and managed according to the trust’s enhanced recovery after surgery (ERAS) pathway. Extubation was performed at the discretion of the treating anaesthetist, and all patients received 28 days of postoperative thromboprophylaxis with low-molecular-weight heparin. Clavien-Dindo postoperative complication severity grading system was utilised for reporting complications after surgery [17].

### Robotic surgical technique

Following induction of general anaesthesia, patients were positioned supine for right-sided resections and in the Lloyd-Davies position for left-sided and rectal resections. All pressure areas were protected, and anti-thromboembolic measures were employed unless contraindicated. Our robotic surgical technique followed standardised institutional practice, with adaptations made for high-BMI patients as outlined below.

Pneumoperitoneum was established using a Veress needle technique. For right-sided colonic resections, patients were placed in a head-down, right-side-up (right side elevation) position. For left-sided colonic and rectal resections, patients were placed in a head-down, left-side-up position (left side elevation). An 8-mm robotic camera port was inserted approximately 2–3 cm above and 2–3 cm lateral to the umbilicus. Additional robotic ports (8 mm) were placed under direct vision to allow appropriate triangulation. A 12-mm robotic port was positioned in the right iliac fossa, between ports two and four. An 8-mm AirSeal port was placed as an assistant port. The Da Vinci Xi robotic system was docked from the patient's left or right side depending on tumour location and surgical plan.

The AirSeal system provides continuous smoke evacuation and stable pneumoperitoneum, which is particularly advantageous in obese patients where increased intra-abdominal pressure and prolonged operative times can otherwise compromise ventilation and visualization. Its valveless design also facilitates frequent instrument exchange by the assistant without loss of pressure, improving operative efficiency in this technically demanding cohort.

In cases where small bowel management proved challenging, particularly in patients with higher BMI and extensive visceral fat, a 4 × 4-cm swab was routinely used to retract and displace the small bowel out of the operative field. This technique facilitated exposure and maintained a clear operative view. A lateral-to-medial approach was often preferred for both colonic and rectal resections to enable early vascular control and efficient dissection along the embryological planes.

In this robotic-assisted approach, the abdominal pressure was maintained between 12–15 mmHg with a CO_2_ flow rate of 40 litres per minute to ensure optimal abdominal distension and visualisation. An additional 5-mm laparoscopic port was placed in the epigastric region to facilitate better control of the small bowel and manage excessive adipose tissue by the surgical assistant. The surgeon utilised robotic instruments, including scissors, a Cadiere grasper, a bipolar fenestrated grasper, and a vessel sealer to precisely divide the mesocolon and omentum. The SureForm robotic stapler, using either a green or blue cartridge, was employed for anastomoses, with two firings in most cases, except in one patient who required only a single firing. As part of the standard procedure, the splenic flexure was routinely mobilised to ensure tension-free anastomosis. All anastomoses were performed intracorporeally using the robotic system to facilitate better exposure and visualisation. Five to 6-cm Pfannenstiel incisions were used for the extraction port and Alexis wound protector used in all cases.

Surgeons predominantly utilised a two right-hand technique to optimise instrument dexterity, facilitating precise dissection and suturing. Standard oncological principles were followed, with high vascular ligation, adequate resection margins, and appropriate lymphadenectomy according to tumour location and preoperative staging. Procedures were performed by either consultant colorectal surgeons or advanced trainees under direct supervision. In our cohort, robotic surgery was performed by high output consultants and did not include procedures performed by surgical trainees. All robotic procedures were performed by high-volume consultant colorectal surgeons; no trainee-performed robotic cases were included.

### Anastomosis technique

All right-sided resections (robotic, laparoscopic, and open where applicable) utilised an intracorporeal side-to-side, isoperistaltic anastomosis. The enterotomy was closed in two layers using a 3/0 V-Loc barbed suture, following standardised institutional practice. There were no differences in anastomotic technique between robotic and laparoscopic approaches; however, in the laparoscopic cohort, all anastomoses except two were performed extracorporeally, and it was left to the discretion of the operating surgeon.

### Statistical analysis

Continuous variables were assessed for distribution. Normally distributed data were presented as mean (standard deviation), while non-normally distributed data were presented as median (interquartile range). Categorical variables were summarised as frequencies and percentages. Categorical variables were compared among robotic, laparoscopic, and open groups using the chi-square test or Fisher’s exact test where appropriate. Continuous variables, summarised as median (range), were compared using the Kruskal-Wallis test with Dunn’s post hoc test and Holm adjustment for multiple comparisons. Given the retrospective design and limited sample size, propensity score matching was not feasible. Outcomes were therefore presented descriptively, with narrative synthesis of perioperative complications by surgical modality. Analyses were conducted using RStudio Team (2023), RStudio: Integrated Development for R. RStudio, PBC, Boston, MA URL http://www.rstudio.com/ [18].

## Results

Between July 2019 and April 2024, 170 patients with a BMI > 30 kg/m^2^ underwent resection for colorectal cancer. Of these, 75 (44.1%) were robotic resections, 60 (35.3%) were laparoscopic, and 35 (20.6%) were open procedures. Table 1 outlines the ASA classification, BMI, and preoperative T stage by surgical technique.

Gender distribution was comparable across groups (*p* = 0.62). Patient age differed significantly between cohorts, with younger patients more frequently undergoing robotic surgery and older patients more likely to receive an open procedure (*p* = 0.02). The distribution of ASA classification did not significantly differ between groups (*p* = 0.21). BMI was significantly lower in the laparoscopic cohort than in the robotic and open cohorts (*p* = 0.03). Preoperative T stage showed borderline significance reflecting a greater proportion of early stage tumours (T1–T2) in the robotic cohort and a higher prevalence of T4 lesions in the open cohort.

Obesity was prevalent among the patients undergoing robotic colorectal resection: 46 (61.3%) were classified as Obesity Class I (BMI 30–35 kg/m^2^) or higher and 30 (40.0%) as Obesity Class II (BMI 35–40 kg/m^2^) or higher, and 27 (36.0%) had severe obesity (BMI > 40 kg/m^2^). The average BMI was 35.8 kg/m^2^, more than one-third of patients had a BMI > 40 and 12 patients (16.0%) > 50, with the highest recorded BMI being 66.1 kg/m^2^. Fifty-six per cent of patients were ASA Grade III or IV. Smoking history was present in 35 (46.7%) patients, 28 (37.3%) were diagnosed with hypertension, and 31 patients (41.3%) had diabetes mellitus.

Patient tumour characteristics, preoperative treatment, and procedures are outlined in Table 2.

**Table 2:** Tumour characteristics, preoperative therapy, and procedures performed in the robotic cohort

Characteristic	Number of patients [*n* (%)]
Location of tumour	
Colon	44 (58.6%)
Rectum	31 (41.3%)
Preoperative chemotherapy ± radiotherapy	
Colon	5 (6.7%)
Rectum	19 (25.3%)
Procedure performed	
Right hemicolectomy	13 (17.3%)
Extended right/subtotal colectomy	5 (6.7%)
High anterior resection	24 (32.0%)
Low anterior resection	13 (17.3%)
Abdominoperineal resection (APER)	18 (24.0%)
Combined procedures (rectum and prostate)	2 (2.7%)

In the robotic cohort, approximately one-third of patients (*n* = 22, 29.3%) received spinal anaesthesia in addition to general anaesthesia and local blocks. Postoperatively, TAP blocks were the predominant analgesic technique, used in 93.3% of cases across both robotic and laparoscopic groups, reflecting the minimally invasive nature of these approaches. The mean total operative time (skin-to-skin, including docking, undocking, and extraction/anastomosis time) for robotic rectal resections was 380 (range 240–450) min and 289 (range 250–410) min for robotic colonic resections. The average Trendelenburg tilt was 20° (range 15–22) with a lateral table tilt of 15° (range 10–20), and the mean head-down duration was 225 (range 120–320) min. Fifty-six patients in the robotic group underwent a primary anastomosis, with six receiving a covering loop ileostomy as part of a low anterior resection. Only one case (1.3%) required conversion to an open procedure, for bleeding control.

In comparison, laparoscopic resections were shorter, with mean operative times of 190 (range 180–250) min for rectal and 165 (range 150–200) min for colonic procedures. Trendelenburg tilt was steeper at 23° (range 10–28), with greater lateral tilt of 18° (range 12–25), and mean head-down duration was 230 (range 110–350) min. By comparison, open resections had mean operative times of 270 (range 250–300) min for rectal and 229 (range 200–250) min for colonic resections. The operative time, degree of Trendelenburg position, and lateral tilt are summarised in Table 3.

**Table 3:** Operative times (time from docking to undocking) and patient positioning in robotic cohort stratified by procedure

Procedure	Number of cases (*n*)	Operating time (min) [median (range)]	Patient positions and tilt angles (°)
Right hemicolectomy	13	120 (100–150)	Head-down 12 (10–20); right side up 15 (10–17)
Extended right/subtotal colectomy	5	180 (150–210)	Head-down 15 (10–20); right then left side up 15–16 (10–20, 12–20) for left colon mobilisation
High anterior resection	24	120 (90–180)	Head-down 15 (12–22); left side up 15 (10–17)
Low anterior resection	13	240 (200–300)	Head-down 17 (12–22); left side up 15 (10–17)
Abdominoperineal resection (APER)	18	140 (110–200)	Head-down 17 (12–22); left side up 15 (10–17)
Combined procedures (rectum and prostate)	2	220 (200–240)	Head-down 17 (12–22); left side up 15 (10–17) (Prostate surgery time not included)

### Anaesthetic outcomes

The intra- and postoperative analgesia strategies used are seen in Table 4, alongside line placements and intra- and postoperative vasopressor use for each surgical approach. General anaesthesia with spinal anaesthesia was most frequently used in the open cohort (62.9%) compared with 35.0% of laparoscopic and 29.3% of robotic procedures (*p* < 0.001). Thoracic epidural analgesia was used exclusively during open surgery (17.1%) and not in the robotic or laparoscopic groups (*p* < 0.001).

**Table 4:** Intra- and postoperative management, including anaesthetic techniques and invasive line approaches stratified by surgical technique

Surgical technique	Robotic [*n* (%)]	Laparoscopic [*n* (%)]	Open [*n* (%)]	Level of significance
Intraoperative analgesia				
General anaesthetic plus spinal	22 (29.3%)	21 (35.0%)	22 (62.9%)	< 0.001
Thoracic epidural analgesia	0 (0.0%)	0 (0.0%)	6 (17.1%)	< 0.001
Postoperative analgesia				
Local anaesthetic wound catheter	0 (0.0%)	0 (0.0%)	24 (68.6%)	< 0.001
TAP block	70 (93.3%)	57 (95.0%)	1 (2.9%)	< 0.001
PCA	73 (97.3%)	59 (98.3%)	35 (100%)	0.340
Lines placed				
Arterial line placed	74 (98.7%)	59 (98.3%)	33 (94.3%)	0.580
Central line placed	4 (5.3%)	7 (11.7%)	34 (97.1%)	< 0.001
Vasopressor use				
Intraoperative	9 (12.0%)	13 (21.7%)	18 (51.4%)	< 0.001
Postoperative	0 (0.0%)	2 (3.3%)	5 (14.3%)	0.004
Surgical outcome				
Resection margin				
R0	74 (98.7%)	58(96.7%)	34(97.1%)	0.827
R1	1(1.3%)	2(3.3%)	1(2.9%)	
R2	0 (0.0%)	0 (0.0%)	0 (0.0%)	
Lymph node yield [median (range)]	17 (12–27)	16 (14–28)	15 (13–28)	0.108

*TAP* transversus abdominis plane (block), *PCA* patient-controlled analgesia

Regarding vascular access, arterial lines were used almost universally across all approaches (98.7% robotic, 98.3% laparoscopic, 94.3% open; *p* = 0.58), whereas central venous lines were placed substantially more often in open surgery (97.1%) than in laparoscopic (11.7%) or robotic cases (5.3%, *p* < 0.001). Vasopressor requirements showed a similar pattern: intraoperative vasopressor use was the lowest in the robotic group (nine patients, 12.0%) compared with higher rates in the laparoscopic (13 patients, 21.7%) and open groups (18 patients, 51.4%, *p* < 0.001), suggesting greater haemodynamic stability with the robotic approach. Postoperatively, vasopressor use was less frequent following minimally invasive surgery, with no cases in the robotic group and only two cases (3.3%) in the laparoscopic group, but more frequent after open resection (four cases, 14.3%, *p* = 0.004), consistent with the greater physiological stress of open procedures.

Postoperative analgesia also varied markedly between cohorts. Local anaesthetic wound catheters were used only following open surgery (68.6%, *p* < 0.001), whereas TAP blocks were routine in the robotic (93.3%) and laparoscopic (95.0%) cohorts but scarcely used in the open group (2.9%, *p* < 0.001). PCA use was similarly high across all groups (97.3% robotic, 98.3% laparoscopic, 100% open) with no significant difference (*p* = 0.34).

Postoperatively, 24 patients (32%) in the robotic group were admitted electively to the critical care unit; 2 (2.7%) required overnight ventilation and were extubated the next morning. The laparoscopic cohort showed similar rates, with 20 patients (33.3%) admitted and 3 (5.0%) requiring overnight ventilation; all were successfully extubated the following morning. In the open cohort, 15 patients (42.9%) required critical care admission, with 4 (11.4%) requiring ventilation overnight; only one was extubated the following morning.

### Surgical outcomes

As shown in Table 5, postoperative pathological T staging was comparable across the robotic, laparoscopic, and open cohorts, with no statistically significant differences (*p* = 0.88).

**Table 5:** Postoperative pathological outcomes by surgical aapproach

Surgical technique	Robotic [*n* (%)]	Laparoscopic [*n* (%)]	Open [*n* (%)]	Level of significance
Pathological T stage (pT)				
pT1	12 (16.0%)	8 (13.3%)	4 (11.4%)	0.880
pT2	18 (24.0%)	15 (25.0%)	7 (20.0%)	
pT3	37 (49.3%)	29 (48.3%)	18 (51.4%)	
pT4	8 (10.7%)	8 (13.3%)	6 (17.1%)	
Resection margin				
R0	74 (98.7%)	58(96.7%)	34(97.1%)	0.827
R1	1(1.3%)	2(3.3%)	1(2.9%)	
R2	0 (0.0%)	0 (0.0%)	0 (0.0%)	
Lymph node yield [median (range)]	17 (12–27)	16 (14–28)	15 (13–28)	0.108
TME grading				
Mesorectal fascia	72 (96.0%)	51 (85.0%)	22 (62.9%)	0.661
Intra-mesorectal fascia	2 (2.7%)	9 (15.0%)	21 (60.0%)	
Muscularis propria	0 (0.0%)	0 (0.0%)	0 (0.0%)	

The complete resection rate was high across all three approaches, with R0 resections achieved in 97.1% of robotic, 96.7% of laparoscopic, and 98.7% of open cases. R1 resections were rare (2.9% robotic, 3.3% laparoscopic, and 1.3% open) and no R2 resections occurred in any cohort, indicating similar margin clearance between groups (*p* = 0.827).

Although the median lymph node yield was highest in the robotic cohort (17, range 12–27), followed by the laparoscopic cohort (16, range 14–28), and lowest in the open cohort (15, range 13–28), this difference did not reach statistical significance (*p* = 0.108).

A similar trend was observed for total mesorectal excision (TME) grading, which was comparable across groups. The highest rate of complete mesorectal excision was achieved in the robotic cohort (96.0%), followed by the laparoscopic (85.0%) and open cohorts (62.9%), with no cases graded as muscularis propria involvement. These differences were not statistically significant (*p* = 0.661).

### Complications

Postoperative complications stratified by surgical technique are presented in Table 6. Perioperative morbidity was lowest in the robotic cohort, with only three Clavien-Dindo grade II–III events (4.0%) and no grade IV–V complications. Conversion to open surgery occurred in only one robotic case (1.3%), compared with eight conversions (15.7%) in the laparoscopic cohort (*p* < 0.001). Anastomotic leaks were recorded in seven robotic patients (9.3%), with anastomotic leaks managed with interventional drainage. Five of the seven anastomosic leaks were treated with interventional radiological (IR) drainage and intravenous antibiotics, and two required laparoscopic wash-out and a defunctioning stoma. No patients required take down of their anastomosis. One patient required washout for a perineal wound collection following an abdominoperineal resection. A single 30-day mortality occurred, and the median length of stay was 7 (IQR 5–10) days.

**Table 6:** Postoperative complications reported according to the Clavien-Dindo classification stratified by surgical techniques

Type of complication	Robotic [*n* (%)]	Laparoscopic [*n* (%)]	Open [*n* (%)]	Level of significance (*p* value)
Conversion to open	1 (1.3%)	8 (15.7%)	NA	< 0.001*
Anastomotic	7 (9.3%)	6 (10.0%)	4 (11.4%)	0.920
Wound infection	0 (0.0%)	2 (3.3%)	5 (14.3%)	0.003
30-Day mortality	1(1.3%)	0 (0.0%)	1 (2.9%)	0.470
Median length of stay (days, IQR)	7 (5–10)	8 (6–14)	10 (7–15)	0.031
Clavien-Dindo grade II–III events	3 (4.0%)	8 (13.3%)	7 (20.0%)	0.040
Clavien-Dindo grade IV–V events	0 (0.0%)	0 (0.0%)	2 (5.7%)	0.008

*NA* not applicable, *IQR* interquartile range, *Clavien-Dindo* Clavien-Dindo postoperative complication severity grading system

*Fisher’s exact test (2-group only; open excluded)

By contrast, the laparoscopic group demonstrated a higher overall complication burden relative to robotic surgery, including six anastomotic leaks (10.0%), two wound infections (3.3%), and eight Clavien-Dindo grade II–III events (13.3%). The median length of stay was 8 (IQR 6–14) days, and no deaths were recorded within 30 days of surgery with no grade IV–V complications were observed.

The open cohort experienced the highest perioperative morbidity. The median hospital stay was longest at 10 (IQR 7–15) days, significantly longer than for minimally invasive approaches (*p* = 0.031). Although higher rates of wound infections (*p* = 0.003) and grade II–III (*p* = 0.040) and IV–V complications were reported in this cohort, the rates of anastomotic leak remained comparable (*p* = 0.92) and one 28-day mortality was observed.

One potential positioning-related complication was identified in the robotic cohort. The patient developed postoperative visual disturbance (non-ischaemic central retinal vein occlusion), and this had returned to normal by the 6-month follow-up. No other positioning-related complications were observed; however, comparable data were not recorded for the laparoscopic or open cohorts.

## Discussion

Robotic-assisted colorectal surgery has become increasingly utilised over the past 2 decades, offering technical advantages over laparoscopic approaches. In obese patients, where pelvic access and visualization are often limited, the stable platform, enhanced three-dimensional vision, and articulating instruments provided by robotic systems are particularly beneficial [19].

In our case series of obese patients, robotic colorectal surgery was demonstrated to be a feasible and safe option, with low conversion rates, a favourable perioperative morbidity profile, and shorter length of stay than open surgery. The conversion rate of 1.3% compares favourably to rates of 5% to 32% reported for laparoscopic colorectal surgery in obese cohorts and is lower than the 7.6% overall robotic conversion rate reported in a recent meta-analysis [20, 21].

The anastomotic leak rate of 9.3% was also similar to the reported leak rates of 10% to 12% reported in prior Western studies evaluating obese patients [22]. Wound infection was lowest in the robotic cohort, aligning with the prior reports where wound morbidity is lowest in minimally invasive surgery. Median length of stay was shorter in the robotic group than in open resection and was comparable to laparoscopic surgery, consistent with evidence that minimally invasive approaches facilitate faster recovery.

Despite these subtle variations in perioperative practice, oncological outcomes were broadly comparable. R0 resection rates exceeded 96% in all groups, with no significant difference in margin status. Taken together, these findings suggest that while surgical and anaesthetic techniques varied widely, oncological clearance was consistently high across modalities.

This conclusion is supported by the comparable preoperative tumour staging across cohorts, as well as the similar postoperative pathological staging and oncological quality metrics, including TME grading and lymph node yield, which collectively demonstrate equivalent early oncological adequacy of resection. Nevertheless, these results should be interpreted with caution given the limitations of the study.

Anaesthetic safety is an equally important consideration in this high-risk population. Although pneumoperitoneum and Trendelenburg positioning can affect respiratory and cardiovascular physiology, colorectal procedures typically require fewer steep head-down tilts (20°) than urological or gynaecological robotic surgery (30 to 40 degrees). This may mitigate risks such as haemodynamic instability, airway oedema, and visual complications associated with steeper Trendelenburg angles [23–25].

Notably, in our cohort, nine (12.0%) patients with a BMI > 40 tolerated > 200 min head down at 20° during their robotic surgery, and this provides some reassurance for those anaesthetising obese patients in the Trendelenburg position. Only one positioning-related complication occurred—a transient non-ischaemic central retinal vein occlusion—which resolved completely by the 6-month follow-up. Although extremely rare, this complication highlights the importance of careful intraoperative monitoring and should be discussed during the consent process for robotic colorectal surgery in high-BMI patients.

Haemodynamic stability was also well maintained across all groups. Intraoperative vasopressor requirements were lowest in the robotic cohort and highest in open procedures, reflecting the higher use of thoracic epidurals. These rates are lower than the 20–40% intraoperative vasopressor use described in laparoscopic colorectal surgery, particularly in patients receiving epidural analgesia, and broadly comparable to the 30% reported in open resections [26]. Postoperatively, vasopressor support was rare across all groups. These figures are consistent with published series where postoperative vasopressor use is < 2% in minimally invasive non-cardiac surgery and between 2 and 5% in open resections, with higher rates in high-risk cases [27]. Taken together, these findings provide reassurance that robotic surgery in obese patients does not confer additional anaesthetic risk, despite prolonged head-down positioning.

Our proactive, multidisciplinary perioperative approach, including routine preoperative assessment and elective critical care admission for those deemed high risk, likely contributed to these favourable outcomes by enabling early detection and management of potential complications. As shown in Table 4*,* wound catheters were used only in open cases (69%), reflecting the predominant use of epidurals in this cohort.

The oncological safety of robotic resection is also an important consideration. In our cohort, the distributions of preoperative tumour stage and early oncological outcomes were comparable across surgical approaches, consistent with large-scale analyses reporting equivalent oncological quality among robotic, laparoscopic, and open colorectal resection.

### Study strengths

This retrospective case series has several strengths. Data were derived from a prospectively maintained cancer registry, reducing collection bias. The cohort included patients with a wide BMI range (up to 66.1 kg/m^2^) and a high proportion of ASA III–IV patients, enhancing the real-world applicability of our findings. Importantly, our comparisons were made against laparoscopic and open resections performed during the same time period at our institution, ensuring contemporaneous benchmarks and reducing bias from historical variation in perioperative practice.

Additionally, the use of a standardised multidisciplinary perioperative pathway reduced variation in anaesthetic and surgical practice. Most robotic resections were performed by experienced, high-volume surgeons, minimising variation from the learning curve or surgical technique. Finally, by reporting both anaesthetic and surgical complications across operative modalities, the study provides a comprehensive assessment of perioperative risk in this high-risk patient population.

### Limitations

Several limitations must be acknowledged. The retrospective design introduces inherent biases related to data collection and patient selection. This was a single-centre study conducted at a high-volume robotic unit, which may limit generalisability to centres with less robotic experience. The relatively small sample size also reduced the ability to detect rare complications or perform robust statistical comparisons. No contemporaneous control group (laparoscopic or open) was available for direct comparison. Longer-term oncological outcomes, such as disease-free and overall survival, were not assessed. Furthermore, we did not capture patient-reported pain scores or functional outcomes, which would provide additional insight into the anaesthetic strategies employed. While allocation to surgical approach in our study was guided by feasibility and patient comorbidities, the retrospective design introduces the potential for selection bias. Future prospective studies should stratify patients by baseline operative risk or incorporate matched cohorts to better control for differences in case complexity across surgical modalities.

Anaesthetic practice was at the discretion of individual anaesthetists rather than standardised, which may have contributed to variation, particularly in postoperative pain scores and analgesia requirements. A key limitation is the absence of a standardised anaesthetic protocol, which introduced unavoidable variation in intraoperative management, postoperative analgesia, and decisions regarding invasive monitoring. This heterogeneity makes it difficult to determine the extent to which anaesthetic factors influenced perioperative outcomes, and it may limit comparability with centres employing protocol-driven pathways. Moreover, variability in consultant-led decision making may have obscured more subtle associations between anaesthetic technique and postoperative respiratory or haemodynamic events, reducing the precision with which causal inferences can be drawn [24].

### Future perspectives

Future studies should include larger multicentre cohorts with prospective designs to validate these findings. Standardised anaesthetic and perioperative protocols, alongside collection of patient-reported outcomes such as pain scores and functional recovery, will be critical to strengthening the evidence base. Incorporating cost analysis would also provide important context given ongoing concerns regarding the resource implications of robotic surgery. Finally, future work should explore the impact of the learning curve and surgeon experience, which may represent important confounders.

## Conclusion

In this single-centre case series, we demonstrate that robotic colorectal surgery is a safe and feasible option for patients with elevated body mass index undergoing resection for colorectal malignancy. With careful multidisciplinary perioperative planning, robotic techniques can mitigate the technical and physiological challenges associated with obesity, achieving low conversion rates, acceptable postoperative morbidity, and favourable early outcomes. Further prospective studies are warranted to validate these findings and assess long-term oncological outcomes.

## Data Availability

The datasets generated and analysed during this study are available from the corresponding author on reasonable request.

## References

[1] Jayne DG, Thorpe HC, Copeland J, Quirke P, Brown JM, Guillou PJ (2010) Five-year follow-up of the Medical Research Council CLASICC trial of laparoscopically assisted versus open surgery for colorectal cancer. Br J Surg 97(11):1638–1645 20629110 10.1002/bjs.7160

[2] van der Pas MHGM, Haglind E, Cuesta MA, Fürst A, Lacy AM, Hop WCJ et al (2013) Laparoscopic versus open surgery for rectal cancer (COLOR II): short-term outcomes of a randomised, phase 3 trial. Lancet Oncol 14(3):210–218 23395398 10.1016/S1470-2045(13)70016-0

[3] Panteleimonitis S, Popeskou S, Harper M, Kandala N, Figueiredo N, Qureshi T et al (2018) Minimally invasive colorectal surgery in the morbid obese: does size really matter? Surg Endosc 32(8):3486–3494 29362912 10.1007/s00464-018-6068-5 PMC6061053

[4] Veldkamp R, Kuhry E, Hop WCJ, Jeekel J, Kazemier G, Bonjer HJ et al (2005) Laparoscopic surgery versus open surgery for colon cancer: short-term outcomes of a randomised trial. Lancet Oncol 6(7):477–484 15992696 10.1016/S1470-2045(05)70221-7

[5] Kang S-B, Park JW, Jeong S-Y, Nam BH, Choi HS, Kim D-W et al (2010) Open versus laparoscopic surgery for mid or low rectal cancer after neoadjuvant chemoradiotherapy (COREAN trial): short-term outcomes of an open-label randomised controlled trial. Lancet Oncol 11(7):637–645 20610322 10.1016/S1470-2045(10)70131-5

[6] Kennedy RH, Francis EA, Wharton R, Blazeby JM, Quirke P, West NP et al (2014) Multicenter randomized controlled trial of conventional versus laparoscopic surgery for colorectal cancer within an enhanced recovery programme: EnROL. J Clin Oncol 32(17):1804–1811 24799480 10.1200/JCO.2013.54.3694

[7] Juo Y-Y, Hyder O, Haider AH, Camp M, Lidor A, Ahuja N (2014) Is minimally invasive colon resection better than traditional approaches? First comprehensive national examination with propensity score matching. JAMA Surg 149(2):177–184 24352653 10.1001/jamasurg.2013.3660 PMC4036435

[8] Bardou M, Barkun AN, Martel M (2013) Obesity and colorectal cancer. Gut 62(6):933–947 23481261 10.1136/gutjnl-2013-304701

[9] Al Kibria GM (2019) Prevalence and factors affecting underweight, overweight and obesity using Asian and World Health Organization cutoffs among adults in Nepal: analysis of the Demographic and Health Survey 2016. Obes Res Clin Pract 13(2):129–136 30718074 10.1016/j.orcp.2019.01.006

[10] Nguyen L, Shanmugan S (2025) A review article: the relationship between obesity and colorectal cancer. Curr Diabetes Rep 25(1):8 10.1007/s11892-024-01556-0 PMC11611961 39621160

[11] Ri M, Aikou S, Seto Y (2018) Obesity as a surgical risk factor. Ann Gastroenterol Surg 2(1):13–21 29863119 10.1002/ags3.12049 PMC5881295

[12] Balentine CJ, Marshall C, Robinson C, Wilks J, Anaya D, Albo D et al (2010) Obese Patients Benefit from Minimally Invasive Colorectal Cancer Surgery. J Surg Res 163(1):29–34 20538294 10.1016/j.jss.2010.03.063

[13] Lascano CA, Kaidar-Person O, Szomstein S, Rosenthal R, Wexner SD (2006) Challenges of laparoscopic colectomy in the obese patient: a review. Am J Surg 192(3):357–365 16920431 10.1016/j.amjsurg.2006.04.011

[14] Leal Ghezzi T, Campos CO (2016) 30 Years of Robotic Surgery. World J Surg 40(10):2550–2557 27177648 10.1007/s00268-016-3543-9

[15] Peters BS, Armijo PR, Krause C, Choudhury SA, Oleynikov D (2018) Review of emerging surgical robotic technology. Surg Endosc 32(4):1636–1655 29442240 10.1007/s00464-018-6079-2

[16] Wysham WZ, Kim KH, Roberts J, Sullivan S, Bhullar S, Roque D et al (2014) Obesity and robotic surgery: associated ventilator indices and perioperative pulmonary complications. J Clin Oncol 32(15_suppl):5596

[17] Dindo D, Demartines N, Clavien P-A (2004) Classification of surgical complications: a new proposal with evaluation in a cohort of 6336 patients and results of a survey. Ann Surg 240(2):205–213 15273542 10.1097/01.sla.0000133083.54934.ae PMC1360123

[18] Medeiros R, Vafi dis J, Smith J, Thomas R (2023) Teaching data analysis tolife scientists using “R” statistical software: challenges, opportunities, and eff ective methods. In: Baumer BS,Çetinkaya-Rundel M, Bray A (eds) *Teaching Tech Together: How to Cultivate a Learning Community for ComputingEducation*. Springer, Cham.10.1007/978-3-031-26010-0_12

[19] Bayraktar O, Aytaç E, Özben V, Atasoy D, Bilgin İA, Erenler Bayraktar İ et al (2018) Does robot overcome obesity-related limitations of minimally invasive rectal surgery for cancer? Surg Laparosc Endosc Percutan Tech 28(1):e8–e11 29252933 10.1097/SLE.0000000000000500

[20] Gahunia S, Wyatt J, Powell SG, Mahdi S, Ahmed S, Altaf K (2025) Robotic-assisted versus laparoscopic surgery for colorectal cancer in high-risk patients: a systematic review and meta-analysis. Tech Coloproctol 29(1):98 40198499 10.1007/s10151-025-03141-3 PMC11978707

[21] Yao Q, Sun Q-N, Ren J, Wang L-H, Wang D-R (2023) Comparison of robotic-assisted versus conventional laparoscopic surgery for mid–low rectal cancer: a systematic review and meta-analysis. J Cancer Res Clin Oncol 149(16):15207–15217 37580404 10.1007/s00432-023-05228-6 PMC11797660

[22] Nugent TS, Kelly ME, Donlon NE, Fahy MR, Larkin JO, McCormick PH et al (2021) Obesity and anastomotic leak rates in colorectal cancer: a meta-analysis. Int J Colorectal Dis 36(9):1819–1829 33796958 10.1007/s00384-021-03909-7

[23] Ferreri FM (2024) Ocular hypertension: new advances/edited by Felicia M. Ferreri. IntechOpen, London

[24] Aceto P, Beretta L, Cariello C, Claroni C, Esposito C, Forastiere EM et al (2019) Joint consensus on anesthesia in urologic and gynecologic robotic surgery: specific issues in management from a task force of the SIAARTI, SIGO, and SIU. Minerva Anestesiol 85(8):871 30938121 10.23736/S0375-9393.19.13360-3

[25] American Society of Anesthesiologists Task Force on Perioperative VisualLoss; North American Neuro-Ophthalmology Society; Society for Neuroscience in Anesthesiology and Critical Care(2019) Practice advisory for perioperative visual loss associated with spine surgery 2019: an updated report by theAmerican Society of Anesthesiologists Task Force on Perioperative Visual Loss, the North American Neuro-Ophthalmology Society, and the Society for Neuroscience in Anesthesiology and Critical Care. Anesthesiology 130(1):12–30. 10.1097/ALN.0000000000002503 10.1097/ALN.0000000000002503 PMC9556164 30531555

[26] Ping C, Lin Q-S, Lin X-Z (2018) Optimal concentration of the transversus abdominis plane block in enhanced recovery after surgery protocols for patients of advanced age undergoing laparoscopic rectal cancer surgery. J Int Med Res 46(11):4437–4446 30111216 10.1177/0300060518790699 PMC6259369

[27] Ramalingam B, Ayotte S, Garcia-Mendez JP, Saric JP, Kožul I, Kovačević J et al (2025) Vasopressor use after noncardiac surgery: an international observational study. Br J Anaesth. 10.1016/j.bja.2025.07.034 10.1016/j.bja.2025.07.034 PMC12799376 40796492

